# Supplementary material for: Somatic burden in Russia during the COVID-19 pandemic
Source: PLoS One. 2023 Mar 10;18(3):e0282345. doi: 10.1371/journal.pone.0282345 (PMC10004591; doi:10.1371/journal.pone.0282345)
Supplement: Attachment — Submitted filename: Response to Reviewers.docx [file pone.0282345.s001.docx]

**Dear Reviewers,**

Thank you for your help in improving our manuscript. We have made the modifications following your suggestions:

**Our reviewers stated that the link for your data was not active. Can you please check and assure that your data is available as you stated in your manuscript.**

Regarding the only point raised by the reviewers, we have verified the link to the dataset in the OSF repository: <https://osf.io/9hw52/> (also available by DOI: <https://10.17605/OSF.IO/9HW52>) - it is indeed correct, and the repository is publicly available without any restrictions.

The Reviewer did not clarify what exactly the issue was when they tried to access the dataset. We tested the link using an anonymous login and found no issues. Please note that the data table cannot be viewed directly within the OSF website interface due to size limitations, but it can be downloaded by clicking on the pictogram next to it.

Because we found no issues with the link, we did not make any modifications to the link or any changes to the manuscript after the previous revision (R1).

**Please review your reference list to ensure that it is complete and correct. If you have cited papers that have been retracted, please include the rationale for doing so in the manuscript text, or remove these references and replace them with relevant current references. Any changes to the reference list should be mentioned in the rebuttal letter that accompanies your revised manuscript. If you need to cite a retracted article, indicate the article’s retracted status in the References list and also include a citation and full reference for the retraction notice.**

Following the journal requirements, we have also verified all the references and ensured that none of the papers we cited were retracted, so there was no need for any changes.

Once again, we thank you for your help and we hope that we have corrected all the inconsistencies and explained all the shortcomings.

With best regards,

Alena Zolotareva on behalf of the co-authors.
